# Reframing organizations in the digital age: A qualitative study exploring institutional social media adoption involving emergency physicians and other researchers

**DOI:** 10.12688/f1000research.73439.2

**Published:** 2021-12-13

**Authors:** Yusuf Yilmaz, Brandon Ruan, Priya Thomas, Victoria Tran, Teresa M. Chan

**Affiliations:** 1 Office of Continuing Professional Development, McMaster University, Hamilton, ON, L8P 1H6, Canada; 2 McMaster Education Research, Innovation, and Theory, McMaster University, Hamilton, ON, L8P 1H6, Canada; 3 Department of Medicine, McMaster University, Hamilton, ON, Canada; 4 Faculty of Medicine, Ege University, Izmir, Turkey; 5 Undergraduate Medical Eduation, University of Toronto, Toronto, ON, Canada; 6 School of Graduate Studies, McMaster University, Hamilton, ON, Canada

**Keywords:** social media, medical education, knowledge translation, organizational change, qualitative study

## Abstract

**Background:** Social media is changing the modern academic landscape; this study sought to explore how organizational structures support or inhibit the harnessing of social media use in academic contexts and knowledge translation.

**Methods:** A qualitative study was conducted using framework analysis based on the Bolman and Deal’s Four-Frame Model—structural, human resources, political and symbolic. The research team used the snowball sampling technique to recruit participants following the completion of each participant’s semi-structured interview. A member check was completed to ensure rigour.

**Results:** 16 social media educators and experts from several countries participated in the study. Study findings showed that within the Structural Frame, participants’ organizations were reported to have with diverse hierarchical structures, ranging hospital-based (strict), education institutional-based and online only groups (malleable). The Human Resources Frame revealed that most participants’ social media organizations operated on unpaid volunteer staff. The training of these staff was primarily via role-modeling and mentorship. Regarding the Political Frame, social media helped participants accumulate scholarly currency and influence within their field of practice. The Symbolic Frame showed a wide range of traditional to non-traditional organizational supports, which interacted with both intrinsic to extrinsic motivation.

**Conclusions:** Bolman and Deal’s Four-Frame Model framework may serve as an effective guideline for academic leaders who wish to strategically implement or enhance social media use into their organizations. The key insights that we have gained from our participants are how new emerging forms of scholarly pursuits can be more effectively enabled or hindered by the attributes of the organization within which these are occurring.

## Introduction

For billions of consumers across the world, social media continues to grow as the epicenter of both information and entertainment. The immense reach and power of social media (broadly described as internet enabled applications that allow users to share/create content and participate in shared networking) is best exemplified during the COVID-19 pandemic, where many took to Facebook, Twitter, and other platforms to discuss, and debate the crisis. ^1^^,^^2^ Alongside these positive applications of social media, rampant usage of social media during the pandemic has also demonstrated its capacity to serve as a vehicle of mis- and dis-information. ^3^^,^^4^

Academic health sciences centers (AHSCs), such as hospitals and universities, are considered disseminators of health information for public health and research. Many are publicly funded and seen as having a social contract for health scholarship and advocacy. ^5^ During the COVID-19 pandemic, AHSCs found themselves ill-prepared to combat this “fake news” ^6^ and the consequences that transpired as a result of its dissemination. ^7^^,^^8^ Some AHSCs simply neglected social media as a tool to combat misinformation, while other AHSCs appeared to actively avoid communicating with their employees, who took to social media to fill the information vacuum- one that was created in the first place by AHSCs. ^9^^,^^10^ There has been a high amount of variability between groups and policies within AHSCs.

Inevitably, this created considerable tension between the AHSCs and their employees, which can be traced to the lack of institutional training such as digital professionalism in alignment with instructional policies and mission statements. ^11^ The consequences of this tension were seen, for instance, when physicians who spoke out on social media about their institution’s lack of preparedness for COVID-19, were terminated. ^12^ Additionally, the absence of social media adoption in AHSCs have resulted in healthcare practitioners creating online-based organizations outside of their professional institution. ^11^^,^^13^^,^^14^ Thus, it is evident that there is misalignment between the expectations of social media usage of individual health professionals’ and their institutions at large. ^15^^,^^16^ This may be due to a lack of compatibility between personal and institutional values with respect to social media. ^17^

There is an opportunity to bring health care organizations and their citizens to bridge this apparent incompatibility in social media usage and public perception. ^18^^,^^19^ As opposed to simply having one small team dedicated within an organization to conduct communications, this can be accomplished by reframing the institution’s organizational framework in the contexts of integrating and adopting social media utilization. One benefit of having organizations in supporting or training staff members’ utilization of social media on the organizational level is that it allows more orientation and role-modeling of professional behavior and conduct on social media. Furthermore, the enabling of physicians and researchers within AHSCs to utilize social media also bring academic benefits for their users, such as forming scholarly communities of practice and increased knowledge translation and research dissemination. ^20^^,^^21^ Knowledge translation is a term described as a process that includes synthesis, dissemination, exchange and application/implementation of knowledge to improve health; some aspects of it has also been described as implementation science or scientific communication/discourse. ^11^ The importance of social media adoption on the institutional level is highlighted in an era of online misinformation. ^22^^,^^23^ The need for reframing organizations in the contexts of social media adoption for knowledge translation will allow AHSCs to better prepare for and combat health misinformation.

Reframing an institution’s organizational framework is a process by which the structure of an organization is aligned with its objectives, with the goal of improving its efficiency and effectiveness. In their book *Reframing Organizations*, Bolman and Deal propose a Four-Frame Model that enables leaders to see organizational issues through four lenses that, altogether, paint a complete picture of the challenge. ^24^ This approach to reframing organizations aims to prevent inefficiencies that may arise when leaders adopt only their own frame of reference, otherwise known as a leader’s *Habitual Frame.* The four frames outlined by Bolman and Deal are the *Structural Frame*, the *Human Resource Frame*, the *Political Frame*, and the *Symbolic Frame*, and they are described in detail in Table 1.

**Table 1.** Bolman and Deal’s Four Frame Model for reframing organizations.

Frame	Metaphor	Central concept	Image of leadership	Leadership challenge
**Structural**	Machine	Efficiency	Social architecture	Attune structure to task, technology, and environment
**Human Resource**	Family	Needs, skills, relationships	Empowerment	Align organization and human needs
**Political**	Jungle	Power, competition	Advocacy	Develop agenda and power base
**Symbolic**	Theatre	Culture, meaning	Inspiration	Create faith, beauty, meaning

Considering the emerging landscape of social media-based scholarship, we sought to harness the conceptual framework proposed by Bolman and Deal to examine how organizational structures emerge or evolve around new forms of scholarship. Specifically, the purpose of this study is to explore how organizational structures support or inhibit the harnessing of social media use in academic contexts and knowledge translation.

## Methods

### Research paradigm

We used quasi-deductive thematic framework analysis approach on the amalgamated perceptions of social media clinician educators and researchers to determine organizational components that fostered the utilization of social media. We used Standards for Reporting Qualitative Research (SRQR) guidelines ^25^ while reporting the results. We chose this technique since we were sensitized to the work of Bolman and Deal previously, and while we did analyze and review the content with a fully inductive approach, we found that our themes mapped well to the framework during conceptual coding. As such, we migrated to a more fully-integrated mapping thereafter to complete the framework analysis. Themes that emerged outside of the framework are presented in addition to those that fit with the framework.

### Research team reflexivity

The research team was comprised of five individuals: three of which used social media professionally in the contexts of scholarly advancement and practice, (YY, PT, TMC) and two who only used it for personal and recreational purposes (VT, BR). Furthermore, the composition of the team consists of a senior lead (TMC) who was an emergency physician. She also was involved in free open access medical education resource dissemination and has a high amount of social media usage. We also involved a postdoctoral fellow (YY) who was an expert in qualitative analysis and social media education. Two researchers (TMC, YY) have publications utilizing qualitative methods. The remaining three members are research assistants trained in qualitative analysis but are neither experts in social media nor knowledge translation (PT, VT, BR). Having a mix of people with different expertise and familiarity was key to ensure that our lead and senior author did not overinterpret the transcripts and bring in their own perspectives and experiences, in keeping with our selected methodology of the framework analysis. Gender composition of the team consisted of three females (TMC, PT, VT) and two males (YY, BR).

### Context

The problem that we sought to answer required seeking experts on social media who had personal and professional backgrounds around knowledge working in AHSCs. As emergency medicine has been a specialty that has employed the use of social media for teaching and learning quite frequently, we did initially focus on this group, but used snowball sampling to go beyond this single specialty. Our initial participants were derived from the field of emergency medicine, as they were likely to have many experiences regarding social media knowledge translation and usage within academic and health institutions based on their extensive online careers. ^26^ Bolman and Deal’s Four-Frame Model guided our study design which examined structural, human resource, political, and symbolic constructs in relation to social media and knowledge translation for organizations. This study is a sub-study that was defined *a priori.* The broader program of research within this domain has been examine and articulate best practices for individual usage of social media for knowledge translation and education. ^11^^,^^21^

### Participant recruitment

We contacted a random selection of 25 individuals from a previously published list of influential physicians ^26^ and contacted them via email or direct messaging on Twitter. The only individual excluded from this random selection was the Principal Investigator (co-author TMC), who is listed in this paper. Based on the literature which identified influential online physicians using a complex set of analytics including network centrality, ^26^ we determined the most influential social media users in emergency medicine and applied a purposive, snowball sampling technique to define our study group, as we have previously used this snowball sampling techniques are used for populations that are ill-defined. ^11^^,^^21^ In this instance, the landscape of social media is constantly evolving with new platforms and new people. Therefore, snowball sampling is an appropriate method to employ to augment previous lists of influencers that may only hold past relevance.

### Ethics

Hamilton Integrated Research Ethics Board granted ethics approval for the study with a reference number of HIREB-#5609.

### Data collection and processing

We elected to perform interviews with our participants since we were interested in individuals’ interaction with their organizations, instead of the interactions between various participants. We developed a semi-structured interview guide to assist with steering the interviews. Our interview guide was developed by two team members (EB, PT) based on the Bolman and Deal’s framework. This initial guide was reviewed by the rest of the team, then piloted with non-participatory individuals to ensure clarity. We did not modify the interview guide since our pilot testers thought that the guide was very clear, and the interviewers felt that we were able to elicit the types of answers required. The interview guide has been fully published ^27^ and is available for full review (see references, citation 27).

Two of the team members (AM, BR) managed the data collection procedures. Each research assistant completed a practice run of data collection with the senior research lead (TMC) preceding data collection. The research team reviewed groups of 2-4 transcripts before proceeding with subsequent interviews and refined the interview questions as necessary. This process enabled the interviews to be increasingly explorative. All the transcription files and their content were anonymized. We assigned a gender-matched name for each file using a random name generator.

We informed our participants about the research, data collection process, and confidentiality procedures. Informed consent was obtained from participants via email prior to the interview, who were then asked to complete a short demographics survey (See the interview guide in the *extended data*). Subsequently, we invited them to be interviewed via Zoom video conferencing software (Zoom Communications Inc., San Jose, CA, USA). We used Zoom’s local audio capturing feature to record all the interviews for transcription purposes. The recordings were transcribed verbatim into Microsoft Word files (Microsoft Corp., Seattle, WA, USA) by a trained medical research transcriptionist (EC).

### Analysis

We followed the five-step process articulated by Ritchie and Spencer ^28^: 1) Familiarization; 2) Identifying a thematic framework; 3) Indexing; 4) Charting; and 5) Mapping and interpretation. Throughout the interview period, our team collaboratively analyzed transcripts. The transcripts were analyzed in batches of 2-4 transcripts at a time using Google Docs (Google Inc., Mountainview, CA, USA) to create our codebook. Key quotations were recorded in the comment feature of Google Docs to augment our codebook. After a period of familiarization, we identified that many of the emergent themes aligned with that of Bolman and Deal’s Four-Frame Model and formally began indexing our themes according to this model. Once we analyzed all transcriptions, our research team had a final charting meeting to refine our coding structure and integrated the findings into Bolman and Deal’s framework. We then asynchronously collaborated to engage in mapping and interpretation of the data.

### Rigor and trustworthiness

The research team ensured the trustworthiness of the study by applying several techniques. All of the members analyzed the qualitative data and served as debriefers throughout the analysis. Concepts that the participants mentioned were identified and investigated at every stage of the analysis as well as the last coding stage. The final themes were confirmed with the frames of Bolman and Deal. While reporting, we used thick descriptions to ensure our participants’ voices were represented to the readers. Participants underwent a member check process by reviewing the results of the study to ensure they resonated with their interviews and experiences. Our results and discussion were refined based on their suggestions to better represent and resonate with their experiences.

## Results

The findings section first describes the demographics of participants, and how our snowball sampling technique captured diverse populations of social media stakeholders and experts. The proceeding section describes the results of our analysis in each element of the Bolman and Deal’s framework. Participant quotations were placed in line to support our interpretations of the data in the contexts of the framework.

### Demographics

A total of 16 participants were interviewed, with an average age of 43.3 (SD = 8.32). The interviews were on average 30.3 minutes long, ranging from 22.5 minutes to 42.1 minutes. This yielded a total of 180 pages of transcripts.

The majority of participants were male (n = 10/16), and many were emergency physicians (n = 12/16). Most of our participants clinically or academically practiced in Western countries: 7 (44%) practiced in the USA; 4 (25%) in Australia, and 3 (19%) in Canada. Furthermore, the roles identified by our sample mostly consisted of clinicians (15.94%), teachers 14 (88%), and researchers (12.75%) which aligned with our intended target audience of capturing the perceptions of clinician educators and researchers working within organizations. We captured: 13 (81%) were affiliated with a national or international organization; 8 (50%) were affiliated with a journal or publication; and 8 (50%) identified not being affiliated with a journal or publication. Our interviewed participants utilized a wide array of social media platforms to disseminate their scholarly activities: 15 (94%) used Twitter; 8 (50%) used ResearchGate; 8 (50%) used Google Scholar; and 8 (50%) had an ORCID profile. Our recruitment process was able to capture a diverse amount of organization affiliations, allowing us to gain multiple perspectives around different organization frameworks. A full summary of participant demographics is outlined in Table 2.

**Table 2.** Demographics of participants (n = 16).

Facet	*f* (%)
**Gender**	
Male	10 (62%)
Female	6 (38%)
**Country of origin**	
USA	7 (44%)
Australia	4 (25%)
Canada	3 (19%)
New Zealand	1 (6%)
Netherlands	1 (6%)
**Roles (Individuals were allowed to indicate multiple roles)**	
Clinician	15 (94%)
Teacher	14 (88%)
Researcher	12 (75%)
Academic leader	7 (44%)
Implementation specialist	4 (25%)
Clinical leader	2 (13%)
**Academic qualifications (Individuals were allowed to indicate multiple qualifications)**	
MBBS/MD/DO	13 (81%)
Master's degree (MBA, MPH, MSc, etc.)	9 (63%)
Fellowship certification	8 (50%)
PhD	1 (6%)
**Academic rank**	
Professor	6 (38%)
Assistant professor	5 (3%)
Instructor or adjunct	1 (6%)
Associate professor	1 (6%)
**Organizational affiliations**	
Affiliated with national or international organizations	13 (81%)
Affiliated with journals of other publications	8 (50%)
Not affiliated with journals or other publications	8 (50%)
Not affiliated with national organizations	3 (19%)
**Social media platform**	
Twitter	15 (94%)
ResearchGate	8 (50%)
Google Scholar	8 (50%)
ORCID	8 (50%)
Facebook	6 (38%)
LinkedIn	6 (38%)
WhatsApp	5 (31%)
Slack	4 (25%)
Instagram	2 (13%)
Reddit	2 (13%)
Academia.edu	2 (13%)

### The structural frame

The structural frame includes the governance, committee structures, policies and procedures, and organizational hierarchies present in institutions. Overall, there were several structural elements within the organizations that tended to incentivize academic work across all contexts. These incentives were described by participants in two ways: financial incentives, and non-financial incentives. Examples of financial incentives included grants and salary support. Most participants noted that they had little-to-no financial incentives. As one participant highlighted: *“The hospital doesn't pay me to use Twitter. They pay me to write papers and to be a doctor. And Twitter … in my view should be an embellishment.”*

Engaging in social media was also be seen as an opportunity cost, drawing scientists and researchers away from currently acceptable forms of scholarly work. However, many participants highlighted substantial nonfinancial incentives which were thought to include opportunities to engage in research and scholarship, to learn about new literature, and to recruit participants into their own scholarship. A participant whose primary incentive for social media use was to familiarize himself with new literature added that it is “ *well worth the investment of time even though it is not compensated*”.

Several structural elements were identified within organizations that supported our participants in their utilization of social media. These structural elements were grouped under three main themes: Hospital-based, Educational institutional-based, and Online organization-based structures.

There was a wide range in elements that were noted within the hospital-based structures, with more being structural barriers rather than enablers. For instance, some of the participants noted that hospitals tended to discourage their social media usage, while others were encouraged to have their own accounts. Mostly, if social media was permitted, it was usually not a high priority for organizations, and consequently participants felt that these roles were secondary to their other official roles. On the other end of the spectrum, one participant noted that their hospital had a fully integrated hospital-based social media team. Such hospitals clearly prioritized this type of communication in a fully structural way by creating infrastructure to support these activities.

Many of our participants also held roles within educational institutions that may or may not have been intertwined with hospitals. Within these organizations, it was felt that structures that helped included the development of transparent policies and guidelines for social media integration into the university. One participant describes his desire for his organization to outline what social media tools they value, why, and how they value social media for promotion:

• *As it is in many institutions it is a rather nebulous concept that the unwritten rule is that it is not highly valued so don't waste your time here with social media. But it is not written explicitly anywhere in the anywhere in the promotions board or criteria. The only thing that is written in black and white at my institution is that if you are going to use social media then avoid politics and avoid slanderous jokes, avoid gender disparity and racial disparity comments. Very explicit statements about what not to do on Twitter but nothing about what to do or how they are going to value it.*

Within this theme was the natural structural alignment between the institutional prerogatives for teaching and the methods by which this teaching could be accomplished via social media structures. Tweet chats or other online conversations or blogs for dissemination of content were seen as easy venues.

**Figure 1.**
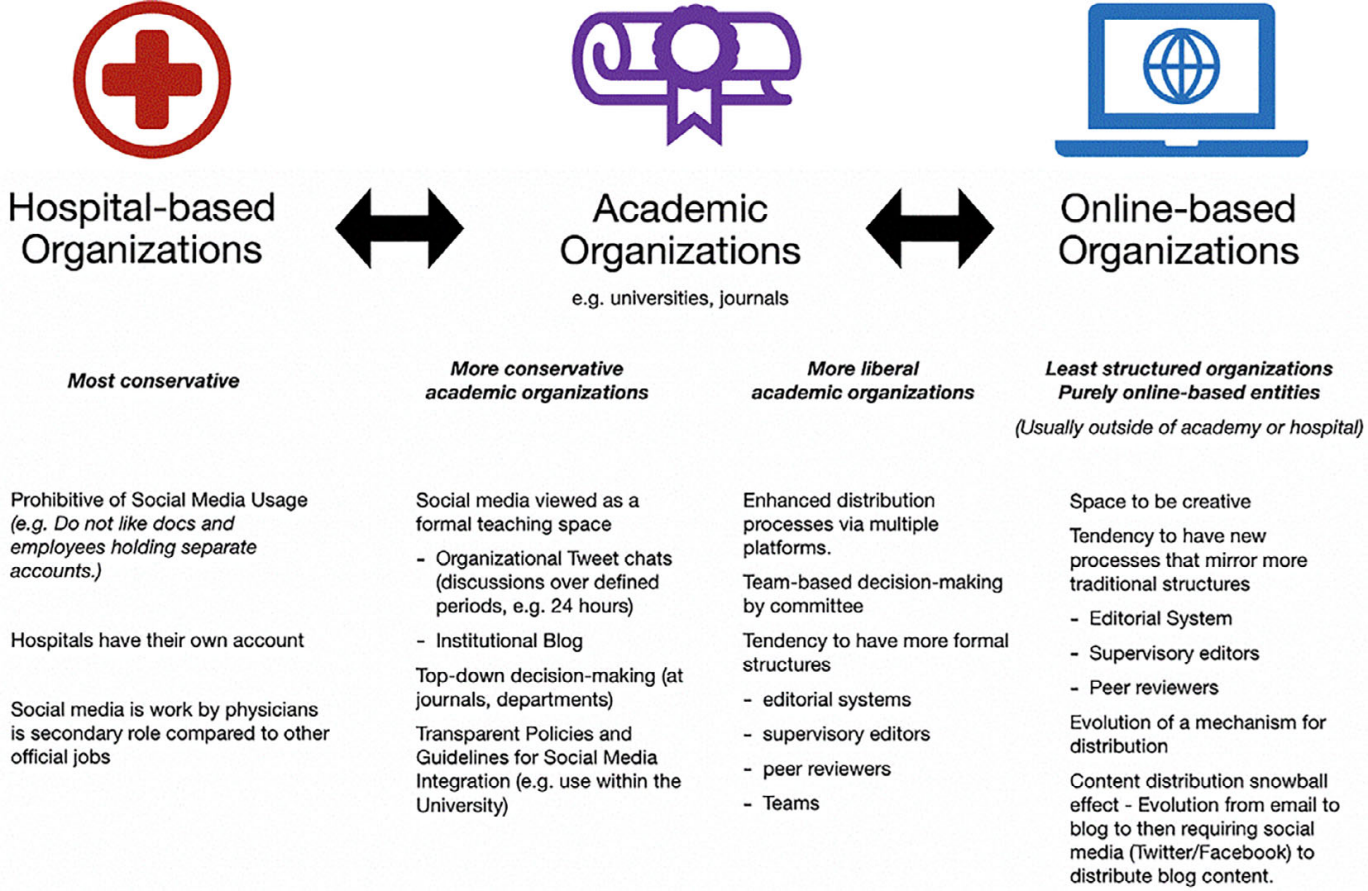
Continuum of structures and policies pertaining to social media.

Online-based organizations tended to have the most non-hierarchical and organic structures where our participants formed groups to engage in content creation (e.g. a blog or podcast). Participants described these structures as spaces to be creative. While some were described to have editorial systems (complete with peer review and editors), most of these organizations were outside the governance of hospitals or educational institutions. Mainly these groups arose as a mechanism to exist outside of formal structures, and yet over time had evolved to take on more structure, increasingly resembling the academic institutions with the evolution of distribution teams and some level of support/hierarchy for authors. Many had also evolved over time to go from more static entities (e.g. blogs) to more media-savvy entities that supported distribution across multiple platforms (i.e. a blog post is distributed across emails, Twitter, and Facebook), which required increased structures to support such processes. Figure 1 depicts the continuum in the level of perceived structures and policies pertaining to social media.

### The human resources frame

**Figure 2.**
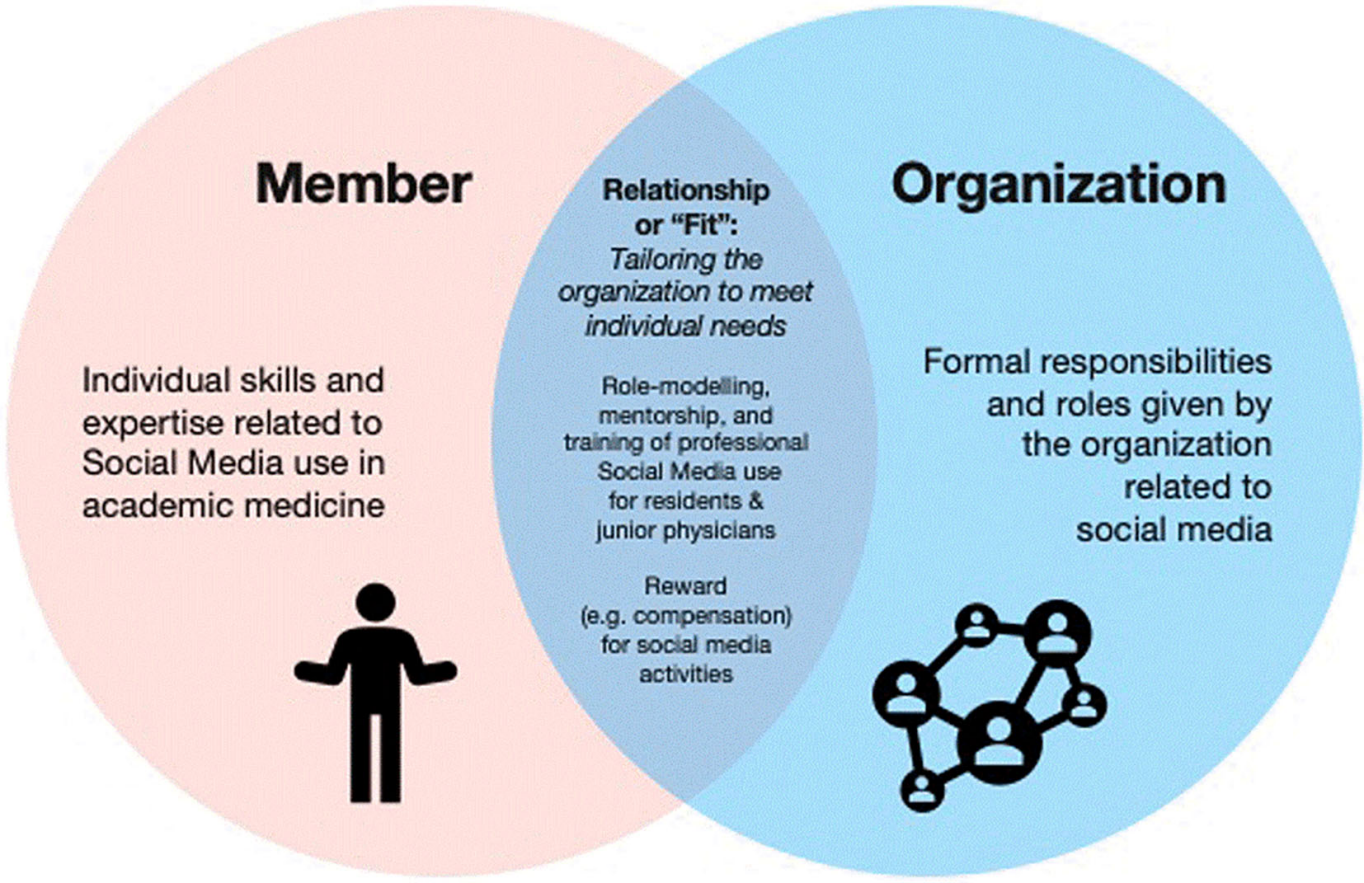
Human resources frame.

The human resources frame describes how an organization fulfills the needs of its members and how the organization allows the worker to express their skills and ideas to create the optimal individual-organizational alignment that benefits both parties (see Figure 2).

***Organizational roles.*** In our interviews, it became clear that organizational roles pertaining to social media usage varied from being informal and volunteer-based, to being delegated with responsibilities, specified tasks, and embedded leadership levels. In most cases, work relating to social media was unpaid and uncompensated. In one participant’s organization, their academic work position did not require blog-writing; however, junior residents were required and expected to write blog posts, with senior residents supporting the blog through administrative tasks and a committee of residents managing the website. The residency blog of their organization has delegated roles for content managers (e.g. “ *putting the graphics up”, “running the website”*) as well as managing editors (e.g. *“administrative tasks”, “emailing people”)* for coordination of content generation within their team of residents.

Another participant described their organization as having *“four levels of involvement”* for social media use: 1) a core executive team of individuals who run the fellowship program, the blog, the social media account, the website, and strategic initiatives; 2) the editors who lead and coordinate a series and set of resources; 3) contributors who help produce podcasts and write blog posts; and finally 4) the supporting staff and junior editors who help upload, copyedit, and perform miscellaneous tasks. Further participants mentioned that they were part of unpaid social media teams populated with individuals who have significant number of followers on Twitter. They are indirectly compensated through the waiving of registration fees for scientific assemblies and unlimited access to various conference sessions to tweet and disseminate information.

***Educational considerations of the human resources themes.*** Training of social media use by organizations through mentorship, role-modelling, and formal education programs within medical school curriculums were mentioned by many participants. These were deemed to be effective methods of supporting an individual’s capacity to learn and empowers members to feel supported and coached in these roles to encourage future participation in social media activities in academic work. These activities and programs were an example of how to address issues arising in the human resources frame such as how to keep individuals informed, involved, committed, and motivated in their work.

A participant noted their start with social media through the role-modelling of colleagues who are experienced social media users, who *“showed [them] that the typical peer reviewed journals don't have as much dissemination as would be optimal”.* With the motivation to promote further dissemination, they learned how to use Twitter and grow an audience, *“specifically to promote the growth of the journal and alt-metrics”.* Another participant added to this notion, and suggested role-modelling through weekly discussion of blogs and podcasts with junior doctors would encourage social media involvement and knowledge on the value of these tools. This participant explains:

• … *what might help particularly for the younger doctors is if for example: we do a blog post of the week and make it easier for younger doctors to access the blog posts and podcasts and to discuss it for example on Tuesday morning after handover. I think something like that would really help younger doctors to get more involved in social media. But also, to get an idea of which blogs and or podcasts are of higher value. And which ones to follow or read.*

One participant described how social media use is being integrated in curriculums for second-year students in the medical education scholarly concentration track; in these social media teaching sessions, students are taught “ *how to utilize [social media] for their professional brand*” and as a way of networking.

### The political frame

**Figure 3.**
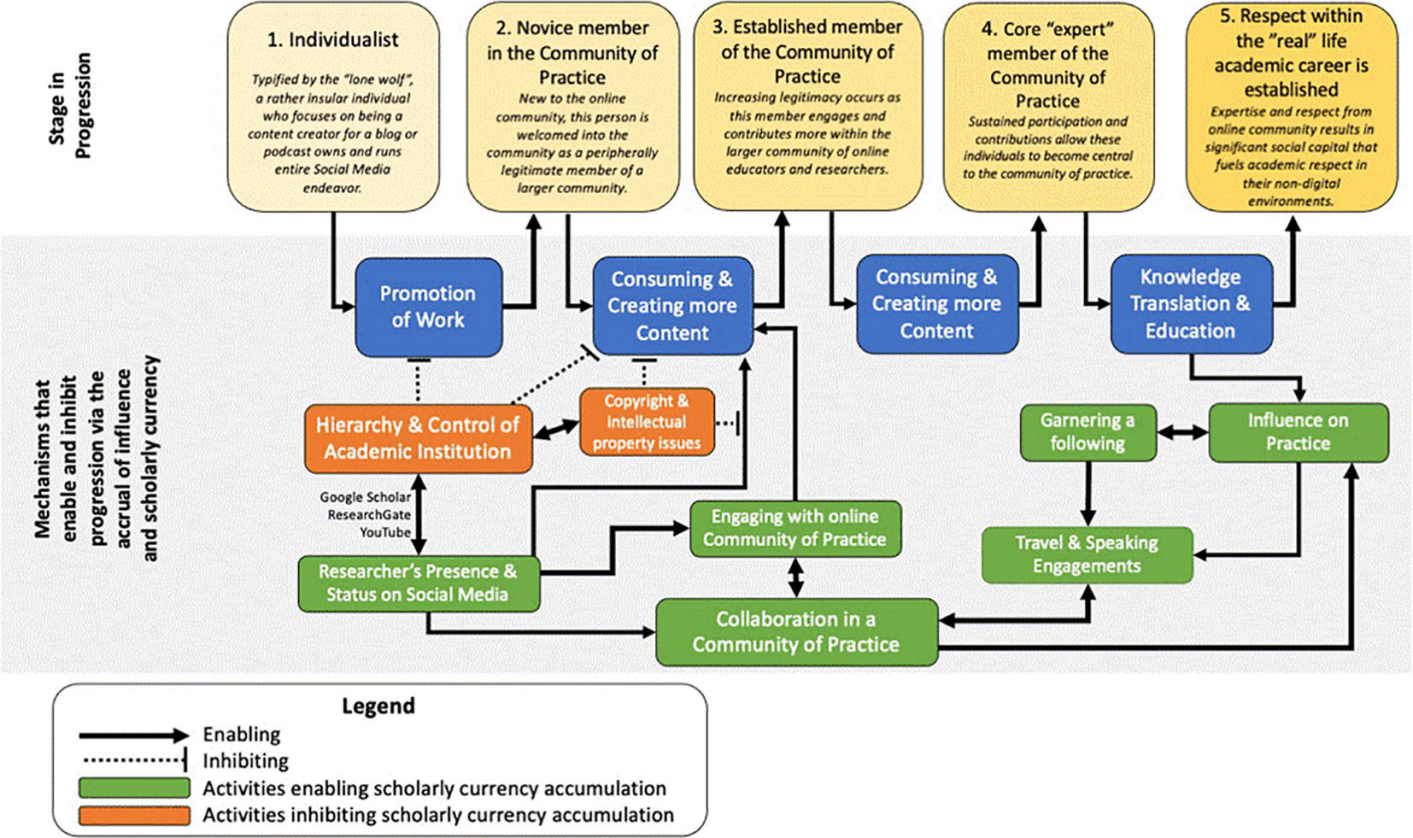
Navigating the political landscape by accruing scholarly currency.

The political frame articulates how various actors within the organization garner and wield influence and power. Our participants described different mechanisms and processes that influenced their behaviors surrounding social media utilization and their organizational influence, which are modeled in Figure 3.

The accumulation of scholarly currency, which we define as the gradual acquisition of political stakeholdership and influence in the respective field of practice, are the drivers for this model. Pointed arrows originating from concepts in green are enablers that promote scholarly currency accumulation, while flat-head arrows originating from concepts in red are inhibitors. Our findings in our model highlight five progressive stages with respect to the online community of practice (CoP) which were be defined further in this section: the “Self” phase, novice member of CoP, intermediate member of CoP, Expert in CoP and acknowledgement in real life.

***Community of Practice (CoP).*** Many participants described engaging, collaborating and networking with a larger network of “scholars” and “communities” on social media, as one interviewee eloquently puts it:

*“Twitter allows you to have a community that you may not have even though you are in a job around similar, like[-minded] people. It allows you to develop an even larger or very strong, connected professional community. It allows you to, you have access to other experts you can learn [from], so something […] is given to you. That is a benefit of Twitter.*”

As this concept frequently emerged in our interviews, we have decided to articulate these entities as the “community of practice (CoP)”, as we felt they were aligned with the concepts originally described by Lave and Wenger. ^29^ A CoP is defined as a common group of people with a shared problem or interest that come together to interact and engage in that shared practice. ^29^ Participants described collaborations within the CoP to serve as the heart when building scholarly currency and academic stakeholdership. Interestingly, by fostering active communities around a given practice, organizations

***The “Self” phase.*** Participants articulated that initial engagement of social media began by developing a digital identity. Participants in this phase carried forward defined research interests and begin to generate forms of content related to their academic work. Utilizing social media’s ability to *“reach a broader audience”* and *“get your message out there”* allowed participants to start building an identity and status as a social media researcher. Participants primarily used Twitter as a “mechanism to promote any of the publications that [they] had at any given time.” Building metrics on other platforms such as Google Scholar or ResearchGate were also ways that contributed to solidifying an online research identity in their field of practice. As the digital identity is in its beginning stages, participants initially engage with CoP in forms of “ *partaking in … conversations and [getting] involved in conversations that other people have started*,” “ *sharing articles that [they] find interesting*” or “ *[commenting] on [the] interpretation of scientific articles.*” Development from this engagement have resulted in early forms of collaborations in some participants, including sharing viewership and collaborating through *“[recording] a podcast with other [podcast organizers].*”

The rate at which online identity manifests was affected by the levels of organizational support received by scholars. Participants who experienced institutional support regarding their social media scholarship found their “ *organization keen to promote and amplify individual Twitter or individual social media outputs from people, particularly when it comes to highlighting publications*,” which assisted in disseminating and promoting their work. Other participants however felt a described “ *lone wolf*” status, where their home institutions did actively not support or engage with their scholarly social media work. These participants, despite valuing institutional support, expressed frustration in receiving lack thereof as one of our participants highlight:

• *I have tried to engage them when there are news stories that are on the mass media that are applicable to our organization. And in that they name our organization for something good that has happened. I tag our organization social media feed—they are on Twitter. Both the hospital and the healthcare system are on Twitter. So, I send them emails saying here is the tweet that I sent. It would be helpful if the organization would retweet this or tag some high-yield folks on this. This is a good story for our organization*… *, [but] there has never, ever been a response, positive, negative, or thank you or anything.*

***Novice members of CoP.*** As the promotion of content continued, types of content and strategies that garnered followers were identified and reproduced, leading to further frequent engagement and acknowledgement with the CoP. At this point, the participants articulated that they transitioned into more online engagement as a social media researcher and has reached the milestone receiving a novice membership in the CoP. Participants described their intention of continuing to engage with their respective CoP by using engagement strategies to promote discussion, as one participant describes: *“When I see new research coming out of any of our journals that I think have a compelling point or merit for the dissemination then I will capture the URL. And send out a tweet with some questions trying to tag a few individuals who I think are in that research field.”*

As production, consumption and engagement continue to build scholarly currency, participants begin to build a “ *network with people who are like-minded [that] increase the chance of collaboration and future grant success*,” which can result in the collaboration of a “ *variety of projects, things like studies, educational endeavors and all sorts of different work.*”

***Intermediate members of CoP.*** Consistent engagement allows further establishment within the CoP, subsequently allowing the third phase of becoming an intermediate member of the CoP. In this phase, the researcher has also established a base audience or target following, and could identify a close group of collaborators. The roles participants took online towards their audience or CoP were diverse. One participant who runs an educational blog as a knowledge dissemination tool for other clinicians described:

• *I spend an awful lot of time writing for an educational blog that I know generates discussion and has positioned me as somebody who influences the discussion around education. And some of the blog post that I am getting are having you know five or six thousand views and comments.*

Other participants established their platform as an education tool for the general public to teach or generate discussions. In one participant’s case, they used their professional expertise in toxicology to “ *teach about drug related topics*” on Twitter. Utilizing the network and following now built, the previous transition mechanisms from stages 2 to 3 were continually repeated to generate scholarly currency.

***Expert members of CoP.*** When enough scholarly currency is attained, the individual became recognized by the CoP as an expert, experiencing academic influence that bleeds and translates into the researcher’s identity in real life: “ *[Twitter] allows you to brand yourself as a leader in the field and it helps you to be established at your own institution.”*

These influences also take the form of many scholarly outcomes. For instance, the opportunities from social media scholarship allowed participants “ *to talk at international conferences and get involved in international guidelines.*” Others commented on social media’s ability to bolster faculty development and find international collaborators, as one of these participants highlight:

• *As a [physician] working in a small community normally not the kind of person that would have a big academic career. And it was only through social media a context that I have been invited to, I have written a number of textbook chapters, I have a large number of academic publications I have been invited to like the [American Heart Association] guideline meetings to speak.*

Our findings from our participants also noted one unconventional academic contribution to social media regarding a grant application as they describe:

• *To be honest a reviewer from my grant actually commented on, like, the amount of followers etc. on my blog and podcast as a positive [factor] when they reviewed my grant application. And [they] noted that it was sort of an atypical pathway but also impressive. So even a very non-social media thing like, you know, applying for a grant for something completely unrelated to social media even that sort of helped me out there.*

***Inhibitors of scholarly growth.*** Meanwhile, our participants described some negative influence towards social media usage. Some organizations were anxious to control their message and utilize their hierarchy to do so, as one participant highlights: “ *[Institutions] seem to be very nervous about their employees or the members of the organization using it because they don't really have control over that content. And that seems to make the organizations a little bit nervous.”*

Political constraints of this type within an organization inhibit individuals from progressing through the various stages of their development from individuals to leaders in their academic spheres. Furthermore, this increases the learning curve for researchers unfamiliar with these platforms that are transitioning to use social media to augment their work. Similarly, the political ramifications (e.g. intellectual property concerns and copyright issues) around content generated within their academic or social media roles had an effect on scholarly growth, often deterring individuals from using social media platforms:

• … *Copyright is tough! …There are others that I know that are producing content that they feel a little bit differently about and are having trepidation about the security of that* … *But certainly, privacy and the ownership of content [issues] of varying degrees, so I know that there are people who are very reluctant to venture into the realm just because of those issues.*

### The symbolic frame

The symbolic frame represents the motivations or sense of purpose from members within an organization. This section provides insights on various ways that organizations can use or support social media to promote their citizens’ sense of purpose and promote their organizational mission. Our analysis revealed that participants had different symbolic values for the use and integration of social media. While some participants sought ways to integrate in their practices in traditional organizations, others found non-traditional groups to associate with. They also mentioned the ways of how intrinsic and extrinsic motivation enables them to contribute to the organization values. For example, enjoyment is worth mentioning as highlighted: “ *I would say that I do that journal club for free. I do it as a member of the editorial board of the journal, it is sort of a service that took up a couple of years ago and I like it. I don't get paid for it.”*

On the other hand, some participants mentioned social media as a manifestation of their mission as a traditional aspect. They perceive that the transition to social media can be attributed to people as well as the organization itself depending on the situation.

The symbolic frame also seemed to have impact on either intrinsic or extrinsic motivations of individuals within organizations. Organizations who supported and rewarded their members in non-traditional ways seemed to be fairly better in generating success for those individuals within the social media domain – suggesting that by supporting and rewarding these individuals, they were providing some level of extrinsic motivation.

Others were less motivated by organizational rewards and support. Many of these individuals noted that it was the other people within the organization that were key to their continued participation. The opportunity to engage in forming new organic social relationships between like-minded people in real life were examples of non-traditional methods of fostering intrinsic motivation. One participant underlined the meaning of strong connection with colleagues here:

• … *we do lots together socially. We are [a] fairly tight-knit group and so I would say that three or four times a week we are doing things socially together and there are a couple of ways that comes to be. Either usually it will be like people who are working together so like staff and residents who are working together will after a shift decide to go out and hangout. Or sometimes it’s like third year residents are responsible for the social kind of aspect of our programs so they will organize things. Or we also have a WhatsApp group through which you know somebody will just organize something sort of casually.*

Traditions have a great deal of impact on social media, as participants mentioned. Organizational supports for social media enabled practices in workplace provide variety of options for their employees. For example, alternative communication options or cultivating communities of practices with organization support is found an important extrinsic motivator. This experience is described in this quote:

• *I would say that my department overall is pretty positive on social media. There are a number of faculty members and residents who have … not [had] incredible involvement in social media but are on Twitter, and [they do have] some participation in some blog activities and other activities in the general [free open access medical education] world. … Overall, I think that the attitude is pretty positive for it.*

In more traditional organizations, social media was often deemed as added activity to be engaged in on individual spare time or perhaps as an adjunct to more traditional scholarly pursuits (e.g. to engage in gaining online engagement with one’s research article). Individuals in these organizations were extrinsically motivated to strongly align their social media work with other traditional roles (e.g. journal editor, researcher) that are already valued, so as to better reap the benefits of the organizations’ support. This participant quote highlights this perceived need for alignment:

• *They might perceive that I spend too much time on it which is perhaps true. But I think it is fair to say that Twitter side, I am perceived as having expertise, world-class expertise I mean in my area of research. And I think that Twitter supports that in a way. And I think the university sees that.*

**Figure 4.**
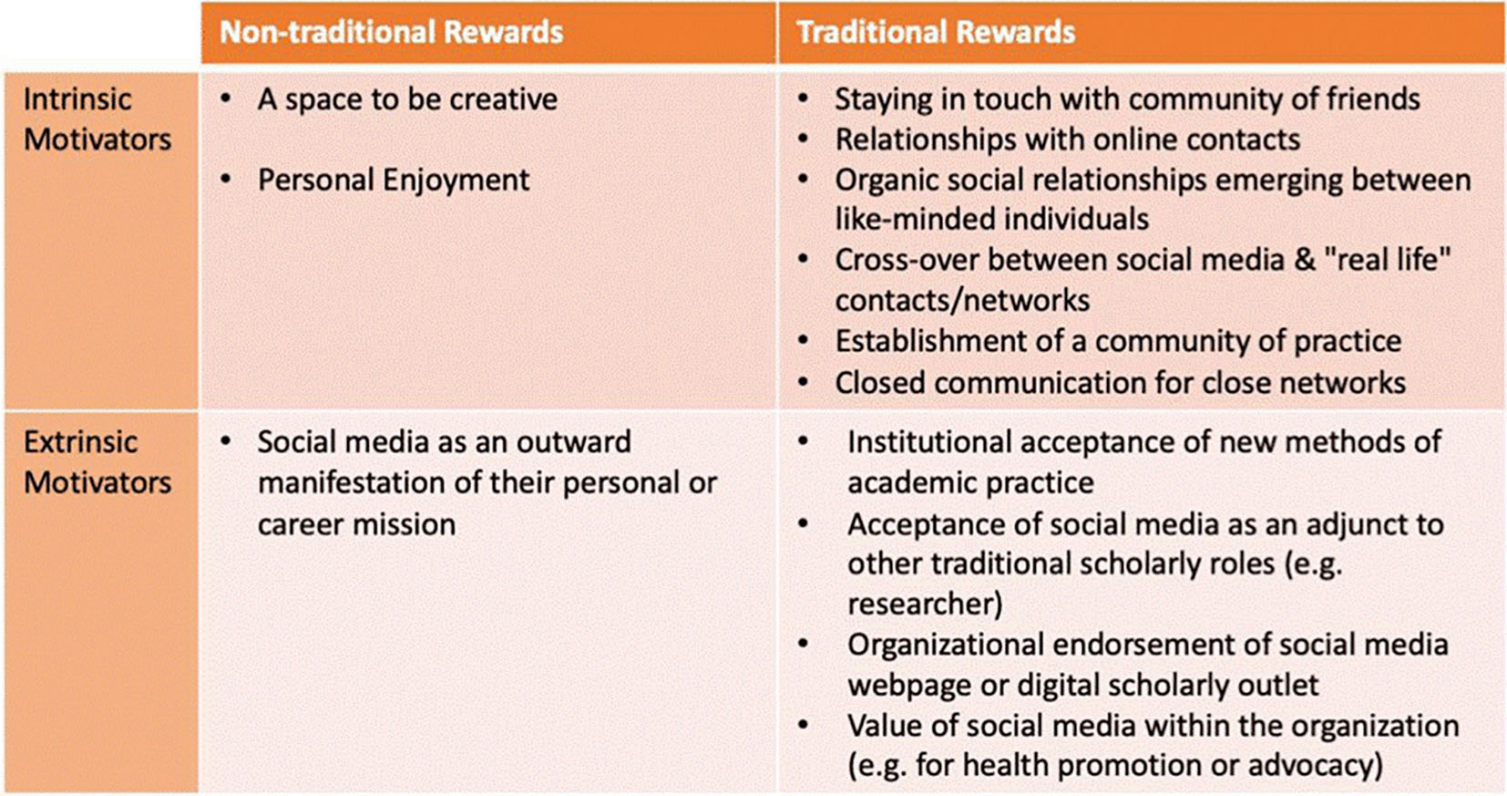
A 2 × 2 table that highlights themes that shed light on how the traditional vs. non-traditional rewards and intrinsic vs. extrinsic motivators intersect.

Figure 4 depicts a 2 × 2 table that highlights how the traditional vs. non-traditional and intrinsic vs. extrinsic motivators intersect.

### The non-organizational personal perspectives

Although the following topics were not considered in the scope of this study, we also found some non-organizational personal perspectives with regards to social media usage that were shared by some respondents. For example, not all respondents were convinced that social media was a useful tool. This might be related to another shared belief that social media promotes interaction without any real emotional investment. Finally, some reported that they are not sure how to use platforms such as Instagram, although they do consider them to be visually appealing.

## Discussion

This study focused on how institutions have been reframing their organizations for using social media, specifically harnessing the lens afforded to us by the Bolman and Deal’s Four-Frame Model. ^24^ Our findings provide insights about how a new form for scholarship (e.g. social media) interacts with hospital-based, academic, and online-only organizations.

Social media has the power to boost organizations’ productivity or visibility ^30^ and applying the four-frame model allows us to identify the structures, human resources, political mechanisms, and symbols that will best enable this. Since our sample had a higher number of emergency physicians than other practitioners, our findings may be particularly salient to individuals who are engaged in leadership within emergency medicine departments. Setting forth a solid structural foundation that acknowledges the existence of social media within an organization is a first step. Subsequently, supporting those structures with adequate human resources to engage in social media opportunities is the next step. Proceeding this, navigating the politics and influence of various forces (such as academic or scholarly credit) is important to bear in mind when aiming to foster social media within health- and academic-based organizations. And finally, symbolic alignment of organizations and individuals can help even greater to build up shared values. Clearly, emerging forms of scholarly work (such as teaching on social media) can be integrated into our existing structures and applying the Four-Frame model may help us to expedite this for other forms of new scholarship.

Bolman and Deal’s Four-Frame Model ^24^ allow us to identify the weaknesses within organizations for any aspect, but in our study, we have used it as a lens to explore social media usage. Our approach of using this framework to analyze social media is a worked example of how one might also investigate the status of any new phenomenon in their organizations. One might similarly have asked clinicians and researchers about how we may use artificial intelligence within medicine through using the Bolman and Deal’s model to examine how institutions are adopting this technology or lack thereof (or not). While this framework is originally meant for leaders who want to change or to build capacity in the organizations, ^24^ it can also be used by those interested in examining emergent phenomena in our organizations.

The key insights that we have gained from our participants are that organizational set-up can enable or hinder new emerging forms of scholarly pursuits, such as social media-based education or scholarship. Although our sampling contained more emergency physicians than other types of healthcare practitioners, we believe the insights from this study will be applicable to other groups within AHSCs as well.

Other change frameworks such as Kotter’s *Eight Steps for Leading Organizational Change* ^31^ or Roger’s *Diffusion of Innovation* ^32^ certainly have some resonance with our present topics. However, Kotter’s framework can be seen more as a playbook for leaders who are implementing an organizational change and does not provide the necessary ingredients to outline how one might ensure they comprehensively prepare their organization for the change, addressed by the way that Bolman and Deal’s Four-Frame Model outlines how one might ensure they comprehensively prepare their organization for the change. Meanwhile, Roger’s model more aptly describes how innovations move throughout society. It has been criticized as a framework for organizations to examine their technology adoption. ^33^ In our present study, one of the key insights is that an individual’s choice to pursue new forms of scholarship may have more to do with the organization perspective on change or innovation, and less to do with their personal attributes. For organizations such as AHSCs to adopt these technologies for the improvement of healthcare, new policies or structures must be created to enable individuals within an organization to thrive – after all, most organizations are groupings of individuals who are connected within a system.

In our study, harnessing the power of Bolman and Deal’s framework maps well to organizational changes as observed by frontline champions of a new technology (i.e. social media). Our findings suggest that others may find it useful for analyzing their organization’s structural, human resources, political, and symbolic components when enacting change. For instance, in the wake of the pandemic many educators are cognizant that there is a need for greater health advocacy, ^34^ health professionals wellness, and equity work. ^35^ Our study suggests that organizations or groups seeking to implement changes to enable these changes may find the Bolman and Deal’s framework useful to review the organizational structures that can enable this type of work to be done. In this study, we have mapped the social media work of emerging digital scholars in emergency medicine and other fields to the Four Frames, others may find this framework similarly useful for mapping the experiences of those in these other new and emerging streams of scholarly work. Strategically, when fostering new lines of scholarship, we propose that our present study shows how relevant the Four Frames can be for improving conditions for those engaging in novel academic work. As seen in our study, when academic leaders attempt to foster new initiatives gaps in the support for these projects can lead to the lack of sustainability for these new types of scholarly pursuits. For instance, if there is a mandate for engaging in a blog, then ensuring there are adequate human resources or budgetary support is imperative.

### Limitations

We worked on eliminating the limitations of the study. Our senior author (TMC) is a social media expert and is highly engaged in organization change strategies as administrator. She was not involved in data collection and was blinded to participants’ identities in this study. While her expertise helped to improve understanding concepts as a leader and social media user, we ensured that her interpretation was aligned with the participants’ views. Participants included international stakeholders who are members of international organizations, but the initial list of social media influencers that we used to initiate the study were mainly emergency physicians resulting in more of this type of practitioner in our study, which may limit the transferability of the findings to other healthcare disciplines. Our participants were also biased towards English-speaking nations (Canada, US, Australia). Potential cultural differences between organizational structures and hierarchies may affect direct transferability of our findings. Our sampling technique may also omit some emerging leaders on social media during the sampling process. Due to the rapidly changing landscape of users and conversations on social media, we may not have included other relevant leaders in our study.

## Conclusions

Social media was originally a user-based platform, but increasingly has a greater role in our academic health sciences organizations for knowledge translation and education. Our findings show that Bolman and Deal's Four-Frame Model may serve an effective guideline for academic leaders who wish to strategically implement or enhance social media into their organizations. Further research is required to elucidate whether enhancing components of the Four-Frame Model may be tactically implemented to improve the capacity of academic institutions to engage in higher quality education or knowledge translation.

## Data availability

### Underlying data

Due to confidentiality reasons our institutional review board does not allow open data sharing of the qualitative data from this study (i.e. the transcripts). For those interested in accessing this dataset, please contact the corresponding author Teresa Chan ( teresa.chan@medportal.ca), who will arrange access to our dataset after institutional protocols including a non-disclosure agreement and/or formal institutional inter-institution agreement are completed.
