# Supplementary material for: Reframing organizations in the digital age: A qualitative study exploring institutional social media adoption involving emergency physicians and other researchers
Source: F1000Res. 2021 Dec 13;10:1048. Originally published 2021 Oct 15. [Version 2] doi: 10.12688/f1000research.73439.2 (PMC8689408; doi:10.12688/f1000research.73439.2)
Supplement: Peer review history

# Peer review: Reviewer response for version 2

**DOI:** 10.5256/f1000research.80075.r108945

**Published:** 2021-12-20

**Authors:** Catherine M. Giroux

**Affiliations:** 1 Faculty of Education, University of Ottawa, Ottawa, ON, Canada

Thank you for the opportunity to review this revised article on social media use within Academic Health Science Centers (AHSCs) based on Bolman and Deal’s Four-Frame model. It is clear that the authors have integrated my previous feedback and I agree with their statement that their revised version presents a stronger argument and rationale throughout the article. In light of the revisions made, I believe this article is ready for indexing.

Is the work clearly and accurately presented and does it cite the current literature?

Yes

If applicable, is the statistical analysis and its interpretation appropriate?

Not applicable

Are all the source data underlying the results available to ensure full reproducibility?

No source data required

Is the study design appropriate and is the work technically sound?

Yes

Are the conclusions drawn adequately supported by the results?

Partly

Are sufficient details of methods and analysis provided to allow replication by others?

Yes

Reviewer Expertise:

Social media; Health Professions Education; Knowledge Translation; Qualitative Research; Mixed Methods Research

I confirm that I have read this submission and believe that I have an appropriate level of expertise to confirm that it is of an acceptable scientific standard.

# Peer review: Reviewer response for version 1

**DOI:** 10.5256/f1000research.77088.r99753

**Published:** 2021-12-02

**Authors:** Catherine M. Giroux

**Affiliations:** 1 Faculty of Education, University of Ottawa, Ottawa, ON, Canada

Thank you for the opportunity to review this interesting and timely qualitative study of social media use within Academic Health Science Centers (AHSCs) based on Bolman and Deal’s Four-Frame model. The article starts with an overview of the role of AHSCs in communicating health-related information and describes social media as one way of doing so. The authors discuss communication challenges that exist between physicians and AHSCs, especially throughout the COVID-19 pandemic and on social media. The authors interviewed 16 emergency physicians to explore how organizational structures emerge or evolve around new forms of scholarship. Specifically, the authors used Bolman and Deal’s framework to explore the hierarchical structures, human resource and training needs, impacts on scholarly currency, and organizational supports for integrating social media within AHSCs. This topic is timely and relevant to a medical education audience.

 I think that this article has merit and should be accepted with reservations. There are a few major and minor points for the authors to address prior to the article being indexed. I will explain my concerns in more detail.

**Major comments:**

1. This study is framed broadly at the institutional level (i.e., academic health sciences centers) but is derived from 16 semi-structured interviews with emergency physicians. Based on the population and sample size, I recommend framing the study as one of emergency physicians’ perceptions of institutional social media adoption since the findings cannot be generalized or easily transferred to specific institutional contexts or to entire institutions.
2. What is missing from both the introduction and discussion section is contextual information about how communications work in AHSCs. For instance, many have designated communications teams that handle social media for the institution. Others may have specific social media policies that are publicly or internally articulated. What implications does this study of emergency physicians’ use of social media and/or perceptions of institutional adoption of social media have for AHSCs with diverse structures and policies? Would the findings of this study be relevant to all groups within an AHSC (e.g., other medical specialties, nursing, rehab, admin, communications)? As written, the study appears to address the barriers and facilitators to physician uptake of social media within AHSCs rather than broad AHSC uptake of social media. 

**Minor Comments:**

**General comments:**

1. Knowledge translation appears in the abstract and throughout the paper but is not defined or operationalized. Including a definition of knowledge translation and explicit connection between social media and knowledge translation would be beneficial.
2. There is inconsistency in the lead author’s initials throughout the paper. Sometimes they include an M and sometimes they do not.
3. Providing a definition of social media would be beneficial; for instance, why is ORCID considered social media in this instance? By some definitions, it might not be.
4. The Findings section is not just a presentation of the findings but rather, includes the authors’ interpretations of the findings. To that end, the authors might wish to consider merging the findings and discussions sections so that they can present their findings and discuss them simultaneously.

**Abstract:**

1. P. 1 - There is some awkward wording in the results section of the abstract. In the structural frame section, the wording “institution types were reported to have with diverse hierarchical structures” is unclear.
2. P. 1 - There is inconsistency in the use of ‘the’ when referring to the frames in the abstract. Including the word ‘the’ before stating each frame (i.e., *the* symbolic frame) is how it is most commonly presented throughout the manuscript.

**Introduction:**

1. P. 3 – The argument in the second paragraph related to AHSCs could be expanded on further for clarity. The current phrasing appears to draw a cause and effect relationship between misinformation and the lack of communication between AHSCs and their employees. Expanding further on why AHSCs were unprepared to tackle misinformation, who the employees referred to are (e.g., communications teams? Physicians?), and who the target audience of communication about misinformation is (i.e., patients/public? Employees?) would be beneficial here.
2. P. 3 - The term ‘online based organizations outside of their institution’ would benefit from definition or examples of what these might be. Operationally defining this term will help the reader differentiate these organizations from formal institutional structures.
3. P. 3 – The sentence “This can be accomplished by reframing the institution’s organizational framework in the contexts of integrating and adopting social media utilization and training towards staff” might benefit from being rephrased or split into two sentences.
4. P. 3 – The sentence “One benefit of having organizations in supporting staff’s utilization of social media on the organizational level will allow more explicit policies for staff that allow professional behaviour and conduct on social media” could also benefit from rephrasing or being split into two sentences.
5. P. 3 – The sentence “The need for reframing organizations in the contexts of social adoption will allow AHSCs to better prepare and combat this” is missing the word ‘media’ following ‘social’. The authors may also consider adding the word ‘for’ following the word ‘prepare’.
6. The overview of Bolman and Deal’s four-frame model is well articulated, especially for those who are unfamiliar with the concept.

**Methods:**

1. P. 3 - Including a short explanation and justification of the quasi-deductive thematic framework analysis approach would be beneficial.
2. P. 3 – SRQR should be written out in full on first use and abbreviated thereafter.
3. P. 4 – the Research Team Reflexivity paragraph switches between past and present tense. Editing for consistency is recommended.
4. P. 4 – The sentence “Furthermore, the composition of the team consists of a senior lead (TMC) who is a medical physician with expertise in qualitative analysis, free open access medical education resource dissemination and social media stakeholdership, a postdoctoral fellow who is an expert in qualitative analysis and social media education” would benefit from being split into two sentences with each sentence reflecting each researcher. The postdoctoral fellow could have their initials identified for consistency since the rest of the team does.
5. P. 4 – The authors indicate that the present study is a sub-study. Additional details regarding the initial inquiry and how this work fits within the overall program of research would be beneficial.
6. P. 4 – A citation supporting the use of snowball sampling for ill-defined populations is recommended.
7. P. 4 – In the first sentence of the Data Collection and Processing section, the apostrophe on the word ‘individuals’ should be placed after the s for a plural possessive (i.e., individuals’).
8. P. 5 – In the Rigour and Trustworthiness section, the sentence “The research team ensured the trustworthiness of the study applying several techniques” would benefit from adding the word ‘by’ before the word ‘applying’.

**Results:**

1. P. 6 – In the Structural Frame section, the sentence “Overall, there were several structural elements within the organizations that tended to incentivize work across all contexts” requires clarification. Is this work related to social media? If not, what kind of work?
2. P. 6 – the sentence “social media can also be seen as a lost opportunity from currently acceptable forms of scholarly activities while spending time on social media” is circular. Why is social media currently not incentivized as a scholarly activity? Alternatively, is the perception that social media is a waste of time?
3. P. 7 – In the second paragraph of the Organizational Roles section, the sentence “further participants were also found to have unpaid social media teams with individuals who have significant number of followers on Twitter” suggests that the participants have their own social media teams. Is this the case?
4. P. 7 – The first sentence of the Educational Considerations section would benefit from being split into two or more separate sentences.
5. The results section alternates between past and present tense. Consistently using the past tense is recommended.
6. P. 9 – The word ‘media’ appears to be missing from the sentence “in these social teaching sessions, students are taught ‘ *how to utilize [social media] for their professional brand’* and as a way of networking”. Media could be inserted between the words ‘social’ and ‘teaching’ in this instance.
7. P. 10 – Adding a brief operational definition of Communities of Practice as conceptualized by Lave & Wenger would be helpful.
8. P. 10 – The words “of which” could be removed from the following sentence for clarity: “The roles of which participants took online towards their audience or CoP were diverse”.
9. P. 11 – In the Inhibitors of Scholarly Growth section: The word ‘which’ could be removed from the following sentence for clarity: “Organizations which were anxious to control their message and utilize their hierarchy to do so…”.
10. P. 12 – The first participant quote is worded in the following way “… not [had] incredible involvement in social media but are on Twitter and do you some participation…” Is this accurate?

**Discussion:**

1. P. 13 - The sentence “While this framework is originally meant for leaders who want to change or to build capacity in the organizations, it can also be used by those interested in examining emergent phenomenon in our organizations” requires a citation.
2. P. 13 – The sentence “The key insights that we have gained from our participants are how new emerging forms of scholarly pursuits can be more effectively enabled or hindered by the attributes of the organization in which they are occurring” appears vague. Including specific examples of the key insights and organizational attributes would be beneficial.
3. P. 13 – The final sentence of the discussion appears incomplete: “As seen in our study, when academic leaders attempt to foster new initiatives, there can be gaps in the support for these endeavours leading to unsustainable”. Unsustainable what?
4. P. 13 – Limitations: Are there any limitations from using a predefined list of social media influencers? Are there any limitations of including only emergency physicians’ perspectives? Are there any geographic limitations regarding who is considered an influencer and is therefore included in the study (i.e., the prevalence of US, Australia, and Canada as compared to how social media is used in institutions elsewhere)? 

Is the work clearly and accurately presented and does it cite the current literature?

Yes

If applicable, is the statistical analysis and its interpretation appropriate?

Not applicable

Are all the source data underlying the results available to ensure full reproducibility?

No source data required

Is the study design appropriate and is the work technically sound?

Yes

Are the conclusions drawn adequately supported by the results?

Partly

Are sufficient details of methods and analysis provided to allow replication by others?

Yes

Reviewer Expertise:

Social media; Health Professions Education; Knowledge Translation; Qualitative Research; Mixed Methods Research

I confirm that I have read this submission and believe that I have an appropriate level of expertise to confirm that it is of an acceptable scientific standard, however I have significant reservations, as outlined above.

# Response

**Published:** 2021-12-02

**Authors:** Teresa Chan

Dear Dr. Giroux,

 Thank you for taking the time to provide us with such a detailed review of our paper. We know that this paper has been enhanced by your time and care, and we are greatly indebted to you for this.

 For the record, we have placed our replies to your thoughtful suggestions alongside your initial reviews. Our replies are indicated by the term **"Reply:" **to differentiate our responses from your initial reviews for future readers.

 Thank you for the opportunity to review this interesting and timely qualitative study of social media use within Academic Health Science Centers (AHSCs) based on Bolman and Deal’s Four-Frame model. The article starts with an overview of the role of AHSCs in communicating health-related information and describes social media as one way of doing so. The authors discuss communication challenges that exist between physicians and AHSCs, especially throughout the COVID-19 pandemic and on social media. The authors interviewed 16 emergency physicians to explore how organizational structures emerge or evolve around new forms of scholarship. Specifically, the authors used Bolman and Deal’s framework to explore the hierarchical structures, human resource and training needs, impacts on scholarly currency, and organizational supports for integrating social media within AHSCs. This topic is timely and relevant to a medical education audience.

**Reply: **Thank you.

 I think that this article has merit and should be accepted with reservations. There are a few major and minor points for the authors to address prior to the article being indexed. I will explain my concerns in more detail.

**Reply: **Thank you for your suggestions.

**Major comments:**

1. This study is framed broadly at the institutional level (i.e., academic health sciences centers) but is derived from 16 semi-structured interviews with emergency physicians. Based on the population and sample size, I recommend framing the study as one of emergency physicians’ perceptions of institutional social media adoption since the findings cannot be generalized or easily transferred to specific institutional contexts or to entire institutions.

**Reply: **Thank you for this comment, as you can see from the first table, our snowball sampling allowed us to start with emergency physicians, but evolved via the snowball sampling to include others. We had physicians from other specialties and also non-physician researchers. We have changed the initial part of the results to explain that 12/16 were emergency physicians. We have changed the title to clarify that this was a study in emergency physicians and other researchers.

  

1. What is missing from both the introduction and discussion section is contextual information about how communications work in AHSCs. For instance, many have designated communications teams that handle social media for the institution. Others may have specific social media policies that are publicly or internally articulated. What implications does this study of emergency physicians’ use of social media and/or perceptions of institutional adoption of social media have for AHSCs with diverse structures and policies? Would the findings of this study be relevant to all groups within an AHSC (e.g., other medical specialties, nursing, rehab, admin, communications)? As written, the study appears to address the barriers and facilitators to physician uptake of social media within AHSCs rather than broad AHSC uptake of social media. 

**Reply: **Thank you for pointing this out. We have altered the initial framing and discussion to highlight this. In the introduction we mention physicians only once pertaining to a specific example, the rest of the framing is around healthcare practitioners more broadly. We have aimed to be both more specific and more broad in our articulation of our intended target audience. We have highlighted how the barriers and facilitators of individuals like our participants engaged in social media are key to broader uptake. We have explained our perspective in this statement within the discussion: “For organizations such as AHSCs to adopt these technologies for the improvement of healthcare, new policies or structures must be created to enable individuals within an organization to thrive – after all, most organizations are groupings of individuals who are connected within a system.”

**Minor Comments:**

**General comments:**

1. Knowledge translation appears in the abstract and throughout the paper but is not defined or operationalized. Including a definition of knowledge translation and explicit connection between social media and knowledge translation would be beneficial.

**Reply: **Thank you for the suggestion. This has been done.

  

1. There is inconsistency in the lead author’s initials throughout the paper. Sometimes they include an M and sometimes they do not.

**Reply: **Thank you for the suggestion. This has been done.

  

1. Providing a definition of social media would be beneficial; for instance, why is ORCID considered social media in this instance? By some definitions, it might not be.

**Reply: **Thank you for the suggestion. This has been done. We have placed our definition in the introduction in parentheses now – “social media (broadly described as internet enabled applications that allow users to share/create content and participate in shared networking)”

  

1. The Findings section is not just a presentation of the findings but rather, includes the authors’ interpretations of the findings. To that end, the authors might wish to consider merging the findings and discussions sections so that they can present their findings and discuss them simultaneously.

**Reply: **The discussion has been kept in this format as we felt it would be important to zoom out again to help readers contextualize our findings. As we are using a framework analysis and a constructivist orientation, our interpretation of the findings are certainly interwoven throughout the paper’s results section.

**Abstract:**

1. P. 1 - There is some awkward wording in the results section of the abstract. In the structural frame section, the wording “institution types were reported to have with diverse hierarchical structures” is unclear.

**Reply: **This has been clarified as best we could.

  

1. P. 1 - There is inconsistency in the use of ‘the’ when referring to the frames in the abstract. Including the word ‘the’ before stating each frame (i.e., *the* symbolic frame) is how it is most commonly presented throughout the manuscript.

**Reply: **This has been done.

**Introduction:**

1. P. 3 – The argument in the second paragraph related to AHSCs could be expanded on further for clarity. The current phrasing appears to draw a cause and effect relationship between misinformation and the lack of communication between AHSCs and their employees. Expanding further on why AHSCs were unprepared to tackle misinformation, who the employees referred to are (e.g., communications teams? Physicians?), and who the target audience of communication about misinformation is (i.e., patients/public? Employees?) would be beneficial here.

**Reply: **This has been clarified. Generally, the literature cited had a broad target audience so the term employees would be quite broadly applicable. We changed the term to “citizens” to make this more clear.

  

1. P. 3 - The term ‘online based organizations outside of their institution’ would benefit from definition or examples of what these might be. Operationally defining this term will help the reader differentiate these organizations from formal institutional structures.

**Reply: **This has been added.

  

1. P. 3 – The sentence “This can be accomplished by reframing the institution’s organizational framework in the contexts of integrating and adopting social media utilization and training towards staff” might benefit from being rephrased or split into two sentences.

**Reply: **We have rephrased this as requested.

  

1. P. 3 – The sentence “One benefit of having organizations in supporting staff’s utilization of social media on the organizational level will allow more explicit policies for staff that allow professional behaviour and conduct on social media” could also benefit from rephrasing or being split into two sentences.

**Reply: **This has been rephrased.

  

1. P. 3 – The sentence “The need for reframing organizations in the contexts of social adoption will allow AHSCs to better prepare and combat this” is missing the word ‘media’ following ‘social’. The authors may also consider adding the word ‘for’ following the word ‘prepare’.

**Reply: **This has been rephrased.

1. The overview of Bolman and Deal’s four-frame model is well articulated, especially for those who are unfamiliar with the concept.

**Reply: **Thank you.

**Methods:**

1. P. 3 - Including a short explanation and justification of the quasi-deductive thematic framework analysis approach would be beneficial.

**Reply: **This has been done.

  

1. P. 3 – SRQR should be written out in full on first use and abbreviated thereafter.

**Reply: **This has been done.

  

1. P. 4 – the Research Team Reflexivity paragraph switches between past and present tense. Editing for consistency is recommended.

**Reply:** This has been corrected.

1. P. 4 – The sentence “Furthermore, the composition of the team consists of a senior lead (TMC) who is a medical physician with expertise in qualitative analysis, free open access medical education resource dissemination and social media stakeholdership, a postdoctoral fellow who is an expert in qualitative analysis and social media education” would benefit from being split into two sentences with each sentence reflecting each researcher. The postdoctoral fellow could have their initials identified for consistency since the rest of the team does.

**Reply: **This has been done.

  

1. P. 4 – The authors indicate that the present study is a sub-study. Additional details regarding the initial inquiry and how this work fits within the overall program of research would be beneficial.

**Reply: **We have done this. We have cited our previous work in the program of research as well.

  

1. P. 4 – A citation supporting the use of snowball sampling for ill-defined populations is recommended.

**Reply: **This has been added. We have cited previous work within our program which has similar techniques.

  

1. P. 4 – In the first sentence of the Data Collection and Processing section, the apostrophe on the word ‘individuals’ should be placed after the s for a plural possessive (i.e., individuals’).

**Reply: **This has been done.

  

1. P. 5 – In the Rigour and Trustworthiness section, the sentence “The research team ensured the trustworthiness of the study applying several techniques” would benefit from adding the word ‘by’ before the word ‘applying’.

**Reply: **Thank you for the careful read.

**Results:**

1. P. 6 – In the Structural Frame section, the sentence “Overall, there were several structural elements within the organizations that tended to incentivize work across all contexts” requires clarification. Is this work related to social media? If not, what kind of work?

**Reply: **We were referring to academic work. This has been clarified.

1. P. 6 – the sentence “social media can also be seen as a lost opportunity from currently acceptable forms of scholarly activities while spending time on social media” is circular. Why is social media currently not incentivized as a scholarly activity? Alternatively, is the perception that social media is a waste of time?

**Reply: **This has been clarified – there is an opportunity cost created by the incentives structures. We have clarified this.

  

1. P. 7 – In the second paragraph of the Organizational Roles section, the sentence “further participants were also found to have unpaid social media teams with individuals who have significant number of followers on Twitter” suggests that the participants have their own social media teams. Is this the case?

**Reply: **We have clarified. It is more that they were part of social media teams.

1. P. 7 – The first sentence of the Educational Considerations section would benefit from being split into two or more separate sentences.

**Reply: **This has been done.

  

1. The results section alternates between past and present tense. Consistently using the past tense is recommended.

**Reply: **We have done our best to adhere to this recommendation. Some sentences are still in present tense because they refer to the Bolman & Deal framework, which we felt was appropriate to keep in present tense.

1. P. 9 – The word ‘media’ appears to be missing from the sentence “in these social teaching sessions, students are taught ‘ *how to utilize [social media] for their professional brand’* and as a way of networking”. Media could be inserted between the words ‘social’ and ‘teaching’ in this instance.

**Reply: **This has been added.

  

1. P. 10 – Adding a brief operational definition of Communities of Practice as conceptualized by Lave & Wenger would be helpful.

**Reply: **This has been added.

  

1. P. 10 – The words “of which” could be removed from the following sentence for clarity: “The roles of which participants took online towards their audience or CoP were diverse”.

**Reply: **This has been deleted.

1. P. 11 – In the Inhibitors of Scholarly Growth section: The word ‘which’ could be removed from the following sentence for clarity: “Organizations which were anxious to control their message and utilize their hierarchy to do so…”.

**Reply: **This has been deleted.

  

1. P. 12 – The first participant quote is worded in the following way “… not [had] incredible involvement in social media but are on Twitter and do you some participation…” Is this accurate?

**Reply: **There was a section that was unclear that resulted in dropping words. We have added bracketed words now for clarity.

**Discussion:**

1. P. 13 - The sentence “While this framework is originally meant for leaders who want to change or to build capacity in the organizations, it can also be used by those interested in examining emergent phenomenon in our organizations” requires a citation.

**Reply: **We have cited Bolman and Deal here.

  

1. P. 13 – The sentence “The key insights that we have gained from our participants are how new emerging forms of scholarly pursuits can be more effectively enabled or hindered by the attributes of the organization in which they are occurring” appears vague. Including specific examples of the key insights and organizational attributes would be beneficial.

**Reply: **We have clarified this statement now.

  

1. P. 13 – The final sentence of the discussion appears incomplete: “As seen in our study, when academic leaders attempt to foster new initiatives, there can be gaps in the support for these endeavours leading to unsustainable”. Unsustainable what?

**Reply: **We have rephrased and added an example for clarity.

1. P. 13 – Limitations: Are there any limitations from using a predefined list of social media influencers? Are there any limitations of including only emergency physicians’ perspectives? Are there any geographic limitations regarding who is considered an influencer and is therefore included in the study (i.e., the prevalence of US, Australia, and Canada as compared to how social media is used in institutions elsewhere)? 

**Reply: **These have been added.

# Peer review: Reviewer response for version 1

**DOI:** 10.5256/f1000research.77088.r97582

**Published:** 2021-11-15

**Authors:** Alice Fornari

**Affiliations:** 1 Department of Science Education, Donald and Barbara Zucker School of Medicine at Hofstra/Northwell, Hempstead, NY, USA

This qualitative study presents data very relevant to medical education today. Social media is a part of daily personal lives and for many has entered their professional lives as a source of colleagueship, resources and recognition of accomplishments. The attempt by the authors to frame the approval, use and extent of institutional support for social media across institutions through semi-structured interviews is definitely out of the box thinking and fills a gap in the literature specific to academic and scholarly activities. Their selection of Bolman and Deal as a leadership and organizational framework is evidenced based outside of academic health centers and is certainly applicable to the academic healthcare institutions and this is evidenced in their results and discussion. Which author made this connection of using this framework should be applauded for connecting an organizational psychology approach to academic healthcare. The introduction fills a gap and captures the reader with a hook. The methods are detailed and follow all "rules" of qualitative research. The results are supported and quotes selected to report engaging are applicable to the original intent of the article. The figures are additive and clarifying and supported by the research process and data collected. 

 I look forward to this article being indexed and shared.

Is the work clearly and accurately presented and does it cite the current literature?

Yes

If applicable, is the statistical analysis and its interpretation appropriate?

Yes

Are all the source data underlying the results available to ensure full reproducibility?

Yes

Is the study design appropriate and is the work technically sound?

Yes

Are the conclusions drawn adequately supported by the results?

Yes

Are sufficient details of methods and analysis provided to allow replication by others?

Yes

Reviewer Expertise:

health professions education, faculty development, mentoring, teaching and learning

I confirm that I have read this submission and believe that I have an appropriate level of expertise to confirm that it is of an acceptable scientific standard.

# Response

**Published:** 2021-11-15

**Authors:** Teresa Chan

Thank you Dr. Fornari for your supportive review of this work.
